# Neurocysticercosis Infection: A Case Report

**DOI:** 10.7759/cureus.36133

**Published:** 2023-03-14

**Authors:** Arjavon T Talebzadeh, Nojan Talebzadeh

**Affiliations:** 1 Surgery, California Northstate University College of Medicine, Sacramento, USA; 2 Surgery, South County Surgery Center, Chula Vista, USA

**Keywords:** host, parasite, transmission, infection, neurocysticerosis

## Abstract

Neurocysticercosis is a condition affecting a significant number of people around the world. The etiology of this condition is a helminth parasite *Taenia solium,* which has a cycle eventually affecting the human host. This condition follows a cycle of human-to-human fecal-oral route and pigs as an intermediate host with transmission to humans. Infected humans can get dissemination of the larva through circulation and spread throughout the body. In this case, the neural tissue was affected. This article will review the neurocysticercosis condition, pathophysiology, mode of transmission, treatment, and complications.

## Introduction

*Taenia solium* is a larval cyst of the tapeworm that affects a significant number of patients around the world. This condition is transmitted from an infected individual with cysticercosis to a new host using a fecal-oral route. In low-income countries, the close proximity of individuals in the same household has a higher incidence of larval transmission. Fecal transmission and proximity of humans to the pigs also lead to secondary infections in pigs. Consumption of undercooked pigs subsequently transmits the tapeworm to a new human host and the cycle continues. In the case of neurocysticercosis, the larvae circulate in the blood and enter the nervous system. The eggs create cysts in different organs leading to a range of conditions from mild to severe and at times lethal [1,2].

Neurocysticercosis could present as an intra-parenchymal or an extra-parenchymal lesion. The location of the lesion can have a significant difference in symptoms, treatment, and prognosis. In the case of intra-parenchymal involvement, the embryos access the gray matter via arteries. Once in place, they grow as a cystic fluid-filled mass surrounded by brain parenchymal tissue. In most patients there is an equilibrium present with no specific symptoms; however, over time, the cyst will damage the surrounding tissue leading to characteristic seizure activity [3]. These cysts may be dormant for years and they may incidentally be detected on imaging [4]. Eventually, the host’s immune system overcomes the homeostasis and the larva dies. The released larva particles generate a significant immune response with various symptoms depending on the location of the cyst. The lesion calcifies and leaves a residual calcification in the brain parenchyma. Most of these cysts have benign prognoses with minimal host symptoms. At times, there may be multiple simultaneous lesions, and this condition is known as cysticercotic encephalitis [5]. 

The extra-parenchymal lesion can involve subarachnoid space, basal cisterns, or ventricles. The enlargement of the cyst in these spaces can obstruct the cerebrospinal fluid flow leading to an increase in the intracranial pressure and possible death from herniations. This report presents a case of a patient who presented to the clinic with an extra-parenchymal lesion which was later diagnosed as neurocysticercosis.

## Case presentation

**Figure 1:**
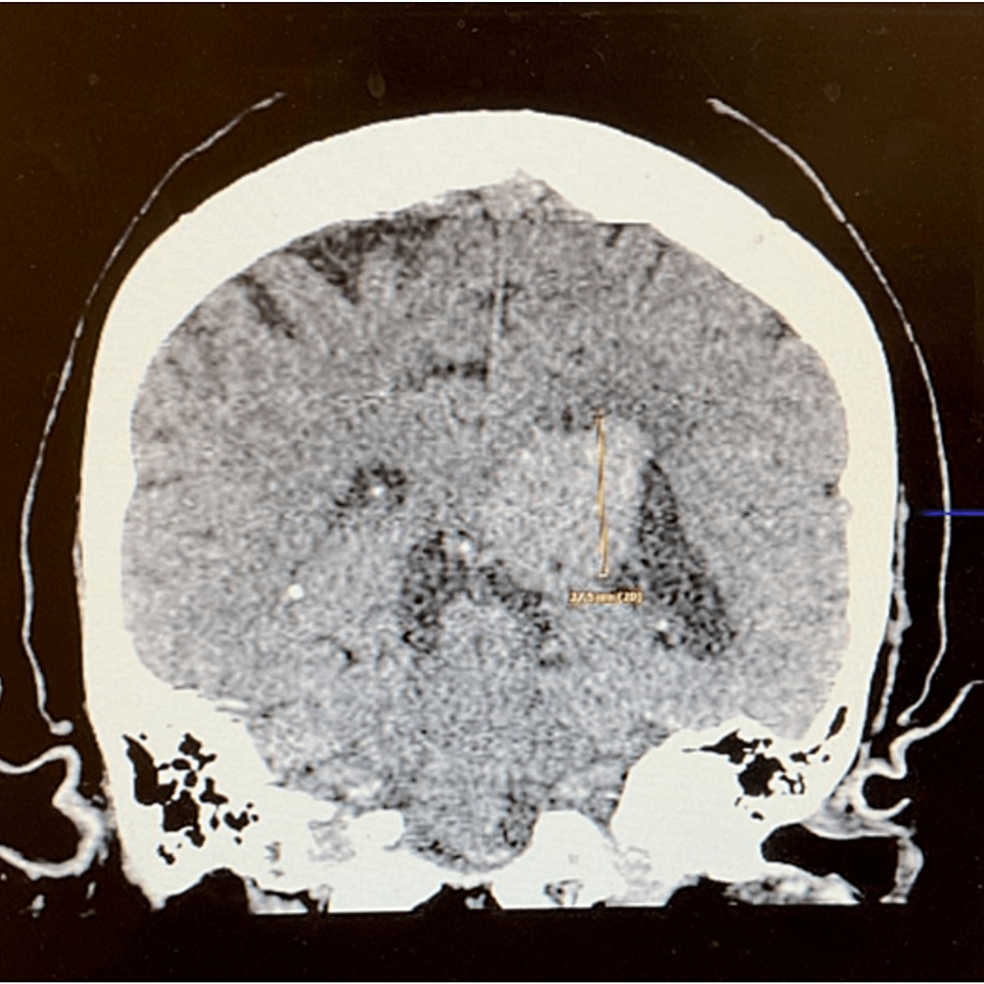
Mass lesion seen in coronal view with signs of calcifications.

**Figure 2:**
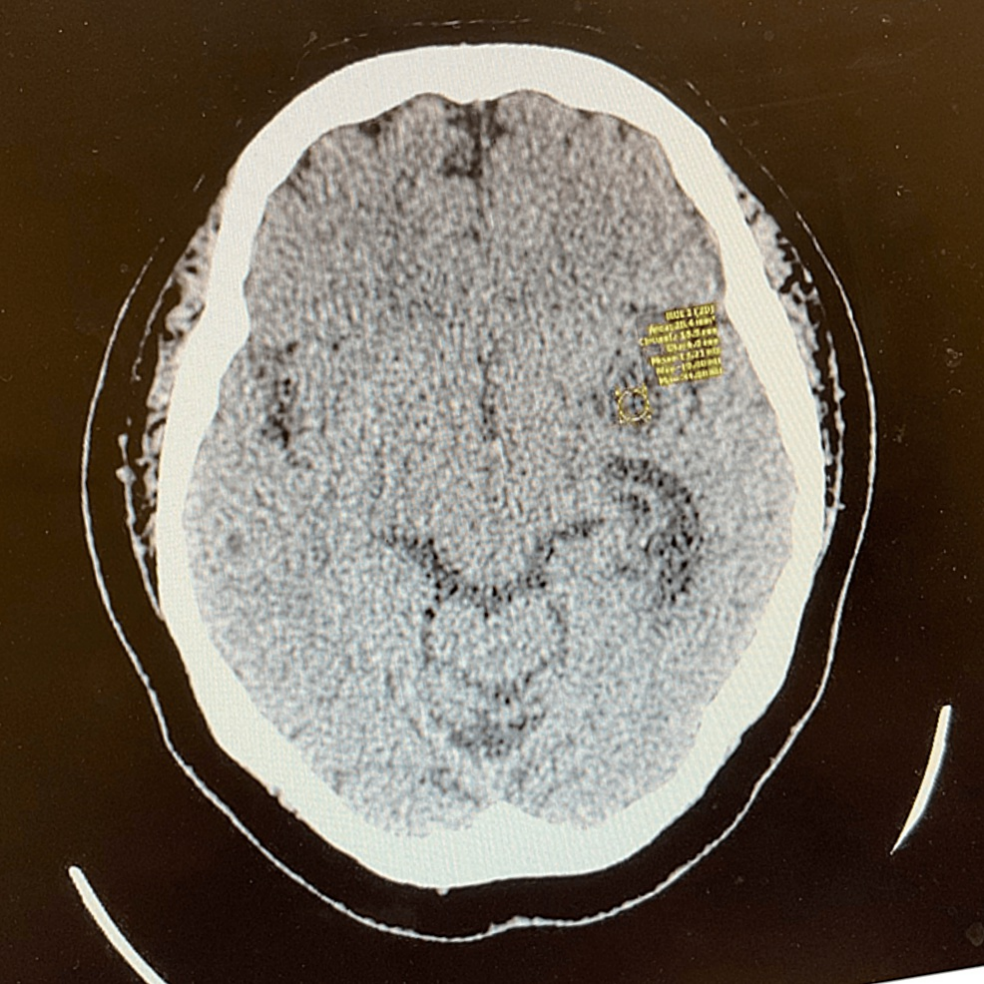
Computed tomography image showing the lesion in axial view.

This is a case of a 65-year-old Peruvian female who presented to the emergency room complaining of a recent headache. Her history is significant for blindness diagnosed secondary to retinitis pigmentosa three years ago. At the time of the presentation, she complained of bilateral vertex headache. The family reported a history of being confused and forgetful at times. She also complained of some vague symptoms of lower left arm numbness. She has no history of upper- or lower-extremity weakness or gait issues. Her past medical history was also significant for type 2 diabetes and a history of hysterectomy. Her medication included amlodipine, atorvastatin, clotrimazole cream, hydrochlorothiazide, indomethacin, sitagliptin, melatonin, and premarin. On presentation, she was afebrile at 37^o^c with stable vital signs and a BMI of 36. She appears older than the stated age. She was awake and alert and in no distress. She communicated in Spanish appropriately and obeyed commands. Blood serology was negative. She was seen by neurosurgery and sent to obtain a computed tomography (CT) scan, which revealed a hyper-dense tumor in the trigone of the left lateral ventricle that measured 30.2 × 17.3 mm. There appeared to be scattered calcifications within the lesion. The mass was blocking the egress of spinal fluid from the left temporal horn and the left occipital horn and the mass was enlarged. However, there was no global mass effect (Figures 1, 2). 

**Figure 3:**
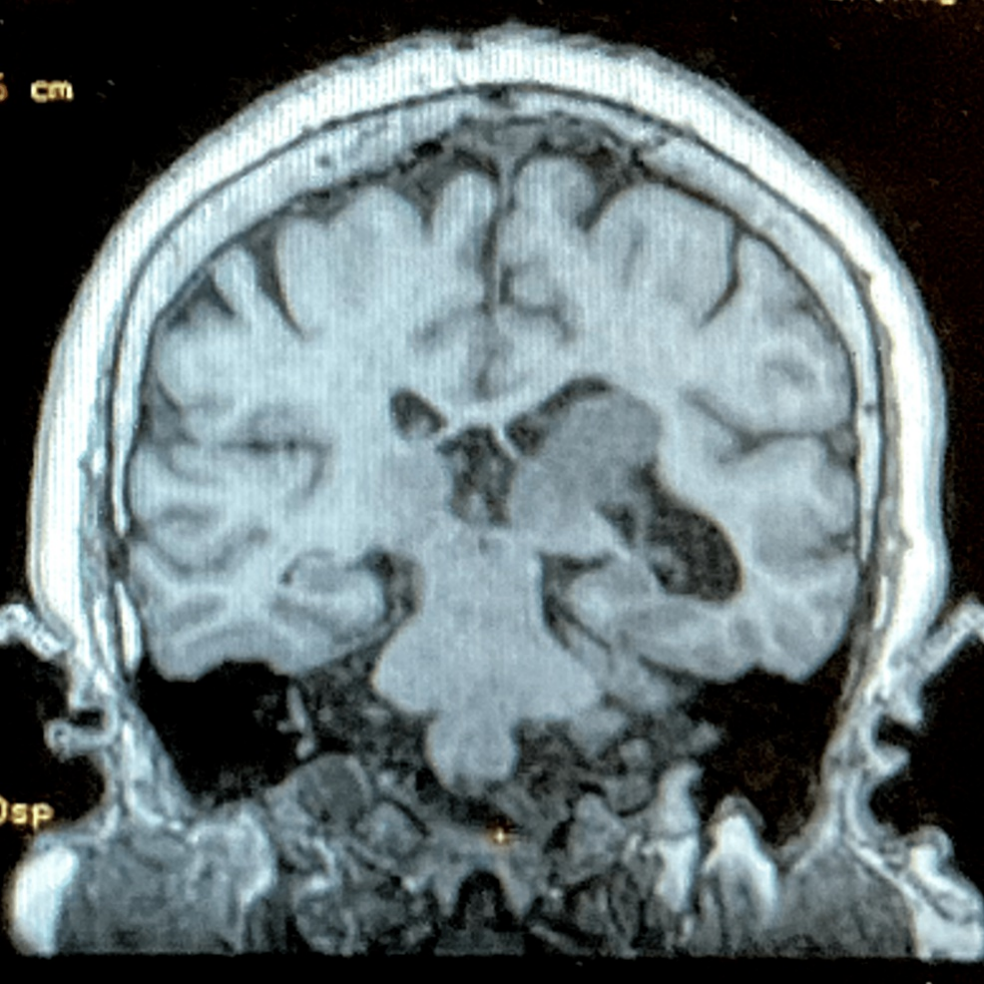
Magnetic resonance image demonstrating the lesion in the ventricle.

She was subsequently sent to get a magnetic resonance imaging (MRI) scan with contrast to better evaluate the lesion. The patient subsequently was traveling and obtained the MRI six months later. The result was significant for an intraventricular tumor on the left trigone (Figure 3).

The differential diagnosis at the time included ependymoma, meningioma, or glioma and possible infections. The subsequent visit to the neurosurgery clinic revealed improvement in the patient’s symptoms although the headache now was localized to the occipital region. Her neurologic examination was otherwise negative [6]. The presence of a mass with calcification and the location of the patient’s birth placed neurocysticercosis high on the list [7]. Serologic testing with enzyme-linked immunosorbent assay (ELISA) was positive. The decision was made to observe the lesion over the next several months due to the location of the lesion. Any signs of growth or impairment would subsequently be treated with medication and a possible endoscopic excision. Subsequent visits revealed the lesion to be stable and planned for a one-year follow-up with an MRI scan [8]. The patient will follow up if there are any new symptoms or if there is a worsening of the headache.

## Discussion

Neurocysticercosis is a common condition seen in third-world countries [9]. There is a very low incidence of this condition in developed countries. Due to the rarity of this lesion in developed countries, practitioners sometimes have low exposure to this disease [10]. Immigration among citizens of different countries has increased the possibility of seeing this condition, and awareness of this disease should be emphasized. The practitioners should be aware of this infection when they are presented with a patient from or traveling to high-risk countries. The location of the lesion is also important due to the range of presentations in these patients. The presentation can range from headache to seizure and finally severe neurologic impairments. Treatment starts with a proper diagnosis. The providers need to be aware of the location and the possible ramification of the treatment. An inactive lesion may be observed and when/if symptoms are present then symptomatic treatment has to be rendered. In those cases treatment options may include antiparasitic medication and if accessible surgical excision [11-13]. 

In 2017, the Infectious Diseases Society of America (IDSA) provided the diagnosis and management guidelines for the treatment of neurocysticercosis [14]. The distinction of the guidelines is based on the symptoms of the patients such as headache, seizures, and signs of neurologic involvement. The general recommendation for intraparenchymal neurocysticercosis includes evaluation for increased intracranial pressure and enzyme-linked immunotransfer blots for confirmation. The patients should be ruled out for tuberculosis prior to anti-inflammatory treatment. The patients should also undergo funduscopic evaluation prior to treatment. Brain MRI and a non-contrast CT scan are also necessary. In the cases of calcified parenchymal neurocysticercosis, the recommendation changes for asymptomatic patients. No anti-inflammatory or antiparasitic medication should be used unless symptoms such as hydrocephalus or seizures are present. MRI with three-dimensional (3D) volumetric sequencing over time is the recommended approach. Antiparasitic therapy namely albendazole and praziquantel has been described in the literature. These medications do kill the larva, however, as a consequence, a severe inflammatory reaction will ensue and it is essential that patients receive anti-inflammatory treatment simultaneously. The anti-inflammatory medication such as corticosteroids will reduce the additional neurologic damage from inflammation [14]. Medical management has been the subject of controversy and it is recommended that patients receiving medical treatment be hospitalized and closely monitored. These medications such as albendazole can have side effects including increased liver enzyme levels; however, liver failure from these medications is rare [14]. It has been suggested that in developing countries mass human chemotherapy may be considered a preventative method to minimize spread. Surgical treatment in case of assessable lesions or endoscopic approaches may also help with some of these lesions [15].

This patient is being followed closely for any worsening of her disease and medical management will be provided if the symptoms worsen. Risks of deleterious side effects have to be evaluated due to the location and inaccessibility of her lesion. Aggressive approach and antibiotic treatment may induce significant inflammation and result in neurologic disruptions. The patient will be following up at the outpatient clinic every six months to assess the prognosis. 

## Conclusions

Neurocysticercosis is a condition that affects third-world countries. Early diagnosis of this disease will give a patient better management options and outcomes. Practitioners in parts of the world that are less exposed to this disease need to keep this condition in their differential looking at the national origin of the patients. This patient is a prime example of a case that would be diagnosed otherwise and treated more aggressively with deleterious effects on the patient's prognosis. In this case, the patient's close follow-up is still indicated to prevent any worsening of her condition, and, as needed, medical management will be provided.
